# Anatomical Museum

**Published:** 1846-12

**Authors:** 


"*Anatomical Museum.*—A New-York correspondent writes that Dr. Beach, of that city, announces for exhibition an extensive collection of specimens of natural and artificial anatomy, the latter being chiefly made of wax, papier-mache, &c., which he has arranged in seven rooms of his own house. In one of those rooms he is careful to publish that he has the *midwifery* department, and in which there are numerous models and preparations illustrating the phenomena of *parturition*, &c., to all which he invites the public, old and *young* indiscriminately, for a few shillings, and the *ladies* especially, for whose special instruction certain hours are set apart exclusively. Lectures on the whole are also promised.

Now to all efforts calculated to diffuse popular knowledge in human anatomy and physiology we have ever been happy to contribute, so far as they are conducted by competent and discreet men, and are limited to such topics as it is every man's and every woman's duty to know, as they have opportunity. But that there are limits to such exhibitions of human structure, and that the proprieties and even the decencies of life are often transcended under the pretext of scientific teaching, has become notorious. Numerous lecturers have been itinerating through the country, with casts, models, drawings, and preparations, which they dignify the name of anatomical museums, their most attractive specimens appertaining to the delicate subjects of conception, gestation, manual and instrumental labor, deformed genitals, &c., the promiscuous exhibition of which has been and continues to be demoralizing and mischievous, especially to the young. The mysteries disclosed to view in the "midwifery department" of Dr. Beach's museum, are not fit objects for young people of either sex to look upon, nor should females be permitted to see such sights, unless they have acquired an age and experience qualifying them for nurses, nor then unless they are destined to be practitioners of midwifery. Nor should any anatomical exhibitions be countenanced by the public, unless public assurances are given that no indelicate or improper features deform the collection, nor unless the respectable character of the exhibiter afford a guarantee to this effect. If under the statutes for the suppression of lewdness, lascivious pictures and obscene books are suppressed by the civil authorities, why should these disgusting exhibitions, so corrupting to public morals, escape the notice of our Grand Juries? That they are pregnant with mischief, cannot be doubted. We may have more to say on this subject hereafter."—*Boston Med. and Surg. Journal.*

